# Using random forest to identify longitudinal predictors of health in a 30-year cohort study

**DOI:** 10.1038/s41598-022-14632-w

**Published:** 2022-06-20

**Authors:** Bette Loef, Albert Wong, Nicole A. H. Janssen, Maciek Strak, Jurriaan Hoekstra, H. Susan J. Picavet, H. C. Hendriek Boshuizen, W. M. Monique Verschuren, Gerrie-Cor M. Herber

**Affiliations:** 1 Center for Nutrition, Prevention and Health Services, National Institute for Public Health and the Environment, P.O. Box 1, 3720 BA Bilthoven, The Netherlands; 2 Wageningen University and Research, Wageningen, The Netherlands; 3 Julius Center for Health Sciences and Primary Care, University Medical Center Utrecht, Utrecht University, Utrecht, The Netherlands

**Keywords:** Biomarkers, Diseases, Epidemiology, Risk factors

## Abstract

Due to the wealth of exposome data from longitudinal cohort studies that is currently available, the need for methods to adequately analyze these data is growing. We propose an approach in which machine learning is used to identify longitudinal exposome-related predictors of health, and illustrate its potential through an application. Our application involves studying the relation between exposome and self-perceived health based on the 30-year running Doetinchem Cohort Study. Random Forest (RF) was used to identify the strongest predictors due to its favorable prediction performance in prior research. The relation between predictors and outcome was visualized with partial dependence and accumulated local effects plots. To facilitate interpretation, exposures were summarized by expressing them as the average exposure and average trend over time. The RF model’s ability to discriminate poor from good self-perceived health was acceptable (Area-Under-the-Curve = 0.707). Nine exposures from different exposome-related domains were largely responsible for the model’s performance, while 87 exposures seemed to contribute little to the performance. Our approach demonstrates that ML can be interpreted more than widely believed, and can be applied to identify important longitudinal predictors of health over the life course in studies with repeated measures of exposure. The approach is context-independent and broadly applicable.

## Introduction

The development of health problems in older age is influenced by a multitude of risk factors to which people are exposed over the life course^1^. With increasing knowledge on risk factors, an ‘exposome approach’ is often advocated, taking into account a broad range of exposures from different domains (i.e. specific/general external, and internal environment) that are repeatedly measured over the life-course^2^. Long-term cohort studies applying this approach can help in identifying predictors of health in older age, which is important for personalized prevention. Since a wealth of data from longitudinal cohort studies is currently available, with each study measuring more aspects of the exposome^3^, there is a need for methods to adequately analyze these large amounts of data.

In trying to predict health based on multiple exposures, we are faced with several challenges. First, the inclusion of many (repeated measurements of) exposures poses considerable challenges, as traditional regression models are generally not well-suited to deal with large numbers of covariates^4^. Second, in such regression models it is often assumed that the relation between each exposure and the outcome is linear in nature and that there are no (or a limited number of prespecified) interactions between exposures. However, these assumptions can often not be verified, and if they are violated, they may potentially lead to wrong conclusions. Nonetheless, these assumptions are frequently ignored or violated, thereby potentially biasing study results^5,6^.

Machine learning (ML), which has been defined as “a family of mathematical modelling techniques that uses a variety of approaches to automatically learn from data, without explicit programming”^7^, offers a solution to deal with limitations of traditional statistical techniques. ML is able to analyze large amounts of data consisting of numerous exposures^8,9^. It can be used to automatically create models that are able to predict the outcome with high accuracy and to identify the most important predicting exposures. In doing so, ML techniques often do not make assumptions on the exact functional form of the model and attempt to learn the model form directly from the data, such that it maximizes prediction accuracy^5^.

While in other research fields the use of ML is already established, within epidemiology and public health research the use of these techniques is still limited, although an increasing number of examples exist where the use of ML has contributed to building prediction models for both diagnosis and prognosis in healthcare research^9,10^. In particular, application of ML to longitudinal data is still in its infancy. The limited use of ML in the field of epidemiology and public health research may be partly because ML models are often considered difficult to grasp. But when used together with methods to facilitate interpretability, there are opportunities for these fields to also incorporate ML techniques to analyze the wealth of data that arise from longitudinal cohort studies^8,9,11,12^. ML can not only be used to generate predictions, but also to identify the strongest predictors for a certain outcome. Here, we propose an approach for this purpose. We illustrate this using an application, in which we identify exposures that were repeatedly measured over the life course that predict (but are not necessarily causally related to) poor self-perceived health. We make suggestions on how to deal with longitudinal exposures, to build a parsimonious prediction model, and to interpret this model. To do so, we chose to use the ML technique random forest^13^, because it is one of the top-performing algorithms in predicting categorical outcomes^14^ and is relatively easy to understand due to its use of decision trees. It is also able to deal with high-dimensional data that may contain non-linear effects and many interactions between covariates, and can be used to rank the most important predictors to gain insight into the resulting prediction model. This ML technique was used on a longitudinal population-based study of adults with up to 6 repeated measurements of exposures over 30 years^15,16^.

## Methods

### Study design and population

**Figure 1:**
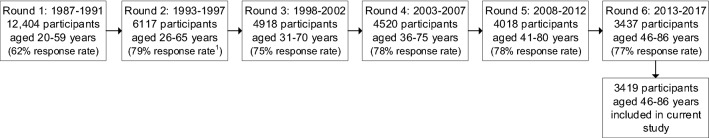
Flowchart of study participants. ^1^ Roughly two-third of the participants from round 1 were randomly selected and re-invited to participate in round 2.

The Doetinchem Cohort Study is a population-based prospective study into the impact of lifestyle and biological risk factors on the health of Dutch adults over the life course^15,16^. In 1987–1991, questionnaires were collected and physical examinations were performed on a random sample of 12,404 inhabitants, aged 20–59 years, from the town of Doetinchem. Of those, 7768 participants were randomly selected and re-invited for participation in the subsequent study rounds every five years (Fig. 1). In the current study, 3419 participants aged 46–85 years at round 6 with complete data on the outcome measure self-perceived health in round 6 were included. Exposures were measured in round 1 through 5. Approval of the Doetinchem Cohort Study was obtained from the external Medical Ethics Committee of The Netherlands Organization for Applied Scientific Research and the University of Utrecht. Informed consent was obtained from all participants. The study was carried out in accordance with the standards set by the latest revision of the Declaration of Helsinki.

### Outcome measure

The outcome measure self-perceived health was measured on a 5-point Likert scale (excellent; very good; good; fair; poor). For ease of interpretation, this measure was dichotomized into excellent/(very) good vs. poor/fair perceived health.

### Exposures

In this study, many exposures from different domains are taken into account, i.e. an ‘exposome approach’ is applied. The ‘specific’ external environment of this exposome concept^2^ is reflected by self-reported lifestyle exposures (e.g. alcohol use/smoking). The ‘general’ external environment is reflected by environmental exposures. They consist of the physical environment *outside* (air pollution/noise/green space measured using qualified methods^17–20^) and *inside* the participants’ home (self-reported in-house environment), and the social environment (self-reported social support/loneliness). The internal environment includes biological exposures, i.e. anthropometric measures (e.g. BMI/blood pressure) measured by trained staff, exposures measured in blood (cholesterol), and self-reported medication use. Additionally, demographic characteristics (e.g. sex/age/education) were included. All exposures are described in Table 1.

**Table 1:** Overview of demographic, lifestyle, environmental, and biological exposures included in the current study.

Exposure	Label	Round^a^
**Demographic exposures**		
Sex	Male; female	r1
Age	In years	r1–r5
Educational level (highest level of education attained)	Primary education or less; lower vocational education or lower secondary education; intermediate vocational education or higher secondary education; higher vocational education or university	r1–r4
Nationality	Dutch; non-Dutch	r1
Marital status	Single, never married; married; widow/widower; divorced	r1–r5
Household composition	With partner; with partner and children; single-parent household; single household; other household	r2–r5
Working hours	In hours per week	r2–r5
**Lifestyle exposures**		
Alcohol use	No, never; no, I stopped using alcohol; every now and then, but less than 1 glass per week; yes	r1–r5
Number of glasses of alcohol per day	In glasses per day	r1–r5
Smoking status	Smoker; former smoker; never smoker	r1–r5
Number of cigarettes per day	In cigarettes per day	r1–r5
Smoking pack years	In the number of smoking years times the number of packs smoked per day	r1–r5
Occupational physical activity (EPIC Physical Activity Questionnaire (Pols et al. 1997))	Sedentary job; standing job; manual work; heavy manual work; not applicable	r1–r5
Time spent on moderate to vigorous physical activity per week (EPIC Physical Activity Questionnaire (Pols et al. 1997))	< 0.5 h; 0.5–3.5 h; ≥ 3.5 h or more, of which < 2 h vigorous; ≥ 3.5 h, of which ≥ 2 h or more vigorous	r2–r5
Dutch Healthy Diet index 2015 (Looman et al. 2017)	On a scale from 0 to 130 (a higher score indicates higher adherence to the Dutch dietary guidelines)	r2–r4
Number of hours of sleep per day	≤ 5 h; 6 h; 7 h; 8 h; ≥ 9 h	r1–r5
Reproductive cycle status	Male; female, regular cycle; female, irregular cycle; female, pregnant; female, anticontraceptive or hormone use; female, unknown/surgery; female, menopause	r1–r5
**Environmental exposures**		
Total NO_2_ concentration at home address (dispersion models (Velders et al. 2020))^b^	In ug/m^3^	r1–r5
Total PM_2.5_ concentration at home address (dispersion models (Velders et al. 2020))^b^	In ug/m^3^	r1–r5
Total elemental carbon concentration at home address (dispersion models (Velders et al. 2020))^b^	In ug/m^3^	r1–r5
Rail traffic noise levels in 2016 for the entire 24-h period at home address (Standard Model Instrumentation for Noise Assessments (Schreurs et al. 2010))	In dB	r1–r5
Road traffic noise levels in 2016 for the entire 24-h period at home address (Standard Model Instrumentation for Noise Assessments (Schreurs et al. 2010))	In dB	r1–r5
Normalized difference vegetation index in 2010 in buffer 300 m around home address (Landsat 5 Thematic Mapper (United States Geological Service)	On a scale from − 1 to 1 (higher score indicating more greenness)	r1–r5
Normalized difference vegetation index in 2010 in buffer 1000 m around home address (Landsat 5 Thematic Mapper (United States Geological Service))	On a scale from − 1 to 1 (higher score indicating more greenness)	r1–r5
Damp stains in the house in the past two years	Not at all; occasionally; often; always	r2–r3
Mold growth in the house in the past two years	Not at all; occasionally; often; always	r2–r3
Hot water supply in the house	Geyser with drain; geyser without drain; boiler; combi boiler; combination or other	r2–r3
Heat source for cooking	Gas; electric; combination or other	r2–r3
Pet (cat, dog, bird or rodent) in the house	Yes; no, not anymore; no, never	r2–r3
Smoking in the participant's environment	Yes, at home and at work; yes, at home; yes, at work; no	r2–r3
Social support measured by positive social experiences (Van Oostrom et al. 1995)	On a scale from 8 to 32 (higher score indicates more positive experiences)	r1–r3
Social support measured by negative social experiences (Van Oostrom et al. 1995)	On a scale from 8 to 32 (higher score indicates more negative experiences)	r1–r3
Social support measure for elderly (Van Eijk et al. 1994)	On a scale from 12 to 48 (higher score indicates more social support)	r5
Loneliness scale (De Jong-Gierveld et al. 1985)	On a scale from 0 to 11 (higher score indicates more loneliness)	r5
**Biological exposures**		
Body mass index	In kg/m^2^	r1–r5
Waist/hip ratio	Ratio	r2–r5
Waist circumference	In centimeters	r2–r5
Pulse rate	In beats per minute	r1–r5
Systolic pressure	In mm Hg	r1–r5
Diastolic pressure	In mm Hg	r1–r5
Total cholesterol	In mmol/l	r1–r5
HDL cholesterol	In mmol/l	r1–r5
Total/HDL cholesterol ratio	Ratio	r1–r5
Use of high blood pressure medication	Yes; no	r1–r5
Use of cholesterol lowering medication	Yes; no	r1–r5

^a^Measurement rounds during which an exposure was measured (round 1 20–59 years, round 2 26–65 years, round 3 31–70 years, round 4 36–75 years, round 5 41–80 years).

^b^Based on concentration estimates of the year 2000 for round 1–3; the average of the years 2000 and 2010 for round 4; and the year 2010 for round 5.

### Statistical analysis

**Figure 2:**
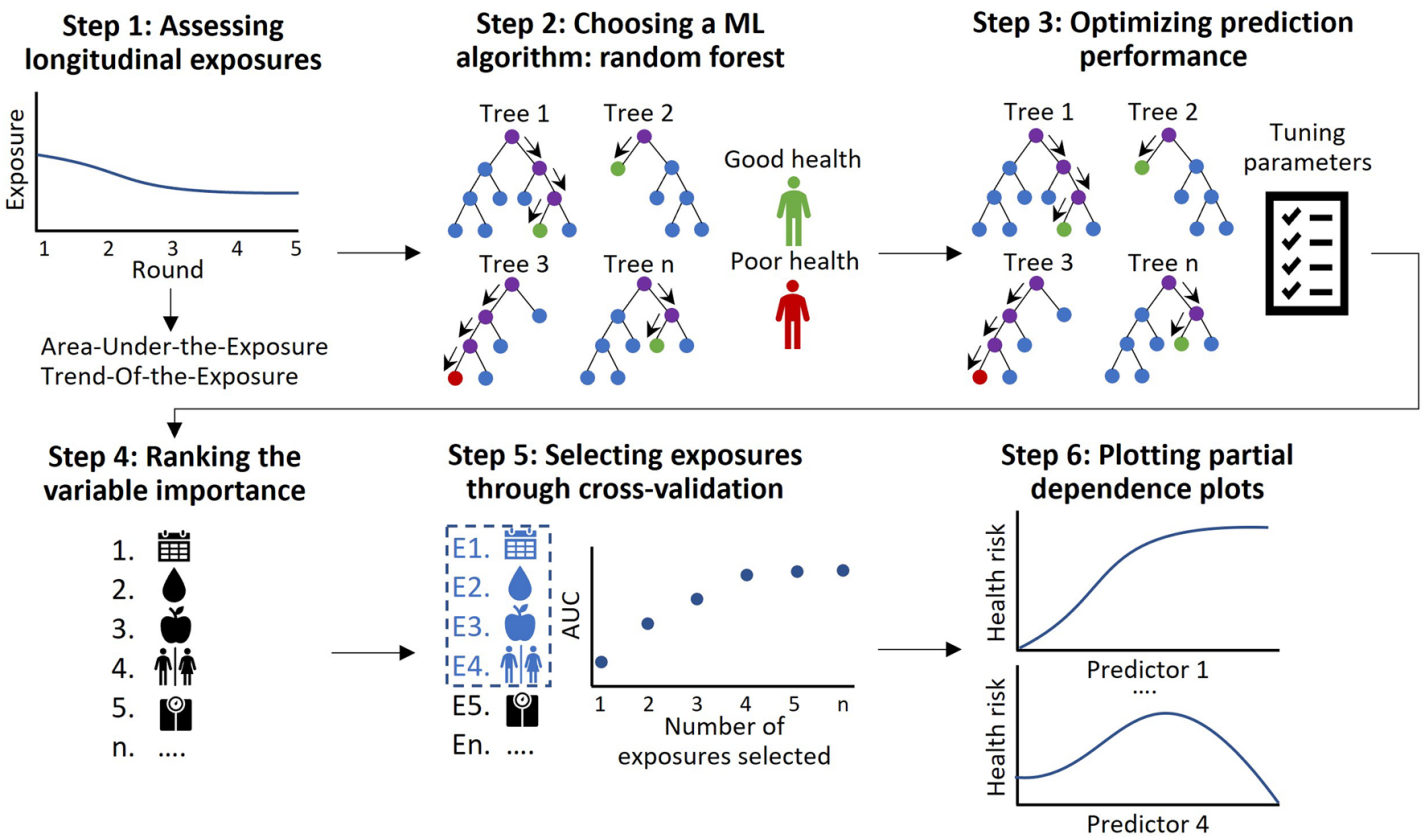
Summary of the six analysis steps.

Statistical analysis consisted of six steps. A summary of these six steps is described below and in Fig. 2. For a full description, we refer to Supplementary Text S1 and Supplementary Text S2.

#### Step 1: assessing longitudinal exposures

To facilitate interpretation, we pre-processed and summarized exposures that were measured during multiple measurement rounds, by introducing the Area-Under-the-Exposure (AUE) and the Trend-of-the-Exposure (TOE). The AUE represents the *average* of the exposure at round 1 through 5. The AUE is computed by plotting observed exposure values against rounds, connecting the values with lines, and determining the *average* area under these lines (continuous exposures) or by calculating the proportion of rounds that the individual occupied a certain state (categorical exposures). The higher the AUE, the higher the prolonged exposure over the life course. The TOE represents the *average trend* in the exposure. It is computed through determining the slope in exposure for each pair of subsequent rounds, and taking the average over that (for continuous exposures) or through determining whether a change from one reference category to another category occurred during the rounds (categorical exposures). A positive value for TOE indicates an upward trend in exposure, whereas a negative value indicates a downward trend. An advantage of this approach is that the AUE and TOE can also be calculated in case of missing values.

#### Step 2: choosing an ML algorithm: random forest

To analyze what longitudinal exposures had the greatest predictive value for self-perceived health, the random forest (RF) algorithm was used^13^. Although many well-suited options are available, RF was chosen due to its consistently good prediction performance^14^. Other advantages of RF are that it: (1) is relatively easy to understand for researchers new to the field of ML due to its use of decision trees; (2) is flexible, as it can be used for both regression and classification tasks and can deal with high-dimensional data that may contain non-linear effects and many interactions between covariates; (3) has a built-in mechanism that can be used to rank the most important predictors and can help the applied practitioner to gain insight into resulting prediction model. This non-parametric ML algorithm consists of an ensemble of decision trees that predict the outcome measure. Within RF, a decision tree is created on a bootstrapped dataset and this step is repeated many times, resulting in a forest of trees. In the current study, the predicted class (good/poor health) by each decision tree was obtained for every individual, and then the proportion of trees that predict poor health was used as the predicted probability of poor health. To determine the prediction performance of the RF algorithm, the Receiver Operating Characteristic (ROC) curve and its Area-Under-the-Curve (AUC), sensitivity, specificity, and accuracy were used. Furthermore, calibration-in-the-large was assessed and a calibration curve was plotted^21^.

#### Step 3: optimizing prediction performance

The tuning parameters of the RF algorithm (i.e. size of random sample of exposures used at each split (*mtry*), number of trees (*ntree*), minimum number of observations in the final nodes (*nodesize*), and maximum number of terminal nodes (*maxnodes*)) were tuned to improve prediction performance^22,23^. In order to choose the optimal parameter settings, we divided the dataset in a random 80% training and 20% test dataset with a similar distribution of the proportions of good/poor perceived health in both datasets. Next, we selected the combination of settings that produced the highest prediction performance on the training dataset with a grid search in combination with five-fold cross-validation (the choice of k in k-fold cross-validation is usually 5 or 10) with R-package caret^24^. Lastly, the model with the optimal settings was used to make predictions on the test dataset and the corresponding ROC curve and AUC were determined.

#### Step 4: ranking the variable importance

One of the primary outcomes of RF is the variable importance ranking, which reflects a ranking of the importance of the exposures in the prediction performance of the RF. For classification (i.e. categorical outcome), the variable importance ranking plot shows a list of ‘most relevant’ variables, that are ranked by mean decrease in accuracy (MDA) that occurs when a particular exposure is permuted randomly in the RF. As the MDA indicates how much accuracy the prediction model losses by removing each exposure, it provides insight into the additive predictive value of a particular exposure in addition to all other exposures. Variables with a large MDA can thus be considered as strong independent predictors of the outcome. The variable importance ranking can be used to investigate and identify associations between exposures and the outcome. We obtained the variable importance ranking by taking the optimal parameter settings and fitting a RF on the entire dataset. In this study, we show the 30 top-ranked exposures in the variable importance.

#### Step 5: selecting exposures through cross-validation

A good prediction model is characterized by its ability to strike a balance between prediction accuracy and parsimony. The variable importance ranking ranks the entire list of features, but does not automatically select the features that together are responsible for the optimal prediction performance. To this end, we considered the number of exposures included in the final model as an additional tuning parameter *q*, and performed a post-hoc cross-validation procedure in which the relationship between *q* and the prediction performance was evaluated, while taking the other tuning parameter values at their previously selected values. Afterwards, the AUC was estimated for each choice of *q*, and plotted against each other. The optimal value for *q* was chosen based on the flattening of the resulting curve.

#### Step 6: plotting partial dependence plots and accumulated local effects plots

The variable importance ranking identifies the most important exposures that predict self-perceived health. However, it does not provide information about the shape of the relation between the exposure and self-perceived health. To visualize this relation, partial dependence plots (PDP)^4^ and accumulated local effects (ALE) plots were produced^25^. These plots illustrate how the prediction of the outcome changes on average when the values of an exposure are changed and while all other exposures are kept constant at their original values. PDPs plot the value of the average predicted outcome on the y-axis against each value of the exposure on the x-axis. ALE plots look at the local effects of an exposure, i.e. the effect is estimated in a subpopulation located in a certain range of the exposure^26^. An advantage of the ALE plots is that they largely avoid extrapolation of the effect at values of the exposure that do not occur in (combination with certain values of another exposure in) the dataset, which is especially a problem when there are highly correlated exposures^25^. However, a consequence of this is that the local effects are only applicable to the specific subpopulation for which it was calculated, and therefore it is difficult to interpret and compare the size of different local effects. In this study, both PDPs and ALE plots were plotted for the number of most important exposures selected through cross-validation. The PDPs provide a general sense of the effect size of each exposure, while the ALE plots were used to check whether the slopes as observed in PDPs are possibly the result of extrapolation issues.

Analyzes were performed using R Version 4.0.2. (http://www.R-project.org/). RF was conducted using the R-package randomForest^27^. The R-package caret was used to tune the RF parameters^24^ and iml was used to plot the ALE plots^26^.

## Results

### Study population (Step 1)

**Figure 3:**
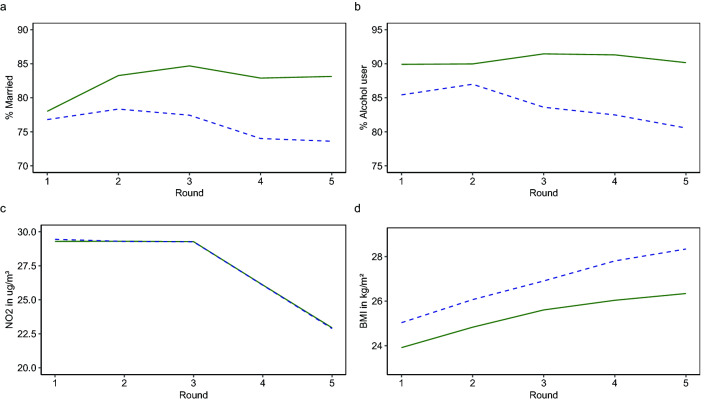
Examples of average trajectories over time of a demographic (**a**), a lifestyle (**b**), an environmental (**c**), and a biological (**d**) exposure for those with good (solid green line) and poor (dashed blue line) perceived health status at round 6.

Of the 3419 participants, 16% reported a poor or fair perceived health at round 6. Table 2 presents a selection of the 96 included exposures, based on the average value (AUE) and trend (TOE) over time of 45 exposures (Supplementary Table S2 presents all exposures) (*Step 1*). Table 2 shows for example that the AUE of the continuous exposure working hours was lower for those with poor versus good (14.9 vs. 22.1 h per week) perceived health, and the TOE was also lower for those with poor versus good (− 1.8 vs. − 1.1 h per week) perceived health, which indicates that a poor perceived health is associated with working on average less hours over time and with a faster decline in the number of hours worked over time. The AUE and TOE of the categorial exposure marital status indicate that those with poor perceived health were less likely to be married over time (76% vs. 82%), and more likely to become widowed or divorced (21% vs. 16%), than those with good perceived health. Table 2 and Supplementary Table S2 also show that there were large differences in other exposures between those with poor and good perceived health. For example, the average BMI (26.9 vs. 25.4 kg/m^2^) and the average trend in BMI (0.8 vs. 0.6 kg/m^2^) over time were higher in those with poor versus good perceived health. Figure 3 presents the average trajectories over time of a demographic/lifestyle/environmental/biological exposure.

**Table 2:** The average value and trend over time of a few selected exposures, stratified by good or poor perceived health status at round 6.

Exposure	Total population (n = 3419)		Good perceived health (n = 2876)		Poor perceived health (n = 543)		*p-value*
	Mean/%	SD/n	Mean/%	SD/n	Mean/%	SD/n	
**Demographic exposures**							
Working hours (in hours per week), AUE	20.9	15.9	22.1	15.6	14.9	16.3	< 0.001
Working hours (in hours per week), TOE	− 1.2	7.5	− 1.1	7.6	− 1.8	7.0	0.040
Marital status (% of the time married), AUE	81	32	82	31	76	37	< 0.001
Marital status (% from married to widowed or divorced), TOE	17	569	16	456	21	113	0.005
**Lifestyle exposures**							
Smoking (in pack years), AUE	9	12	8	11	12	14	< 0.001
Smoking (in pack years), TOE	0.8	2.2	0.8	2.1	1.1	2.9	0.005
Alcohol use (% of the time every now and then or yes), AUE	89	26	90	24	84	31	< 0.001
Alcohol use (% from never user to current user), TOE	9	297	9	251	8	46	0.914
**Environmental exposures**							
NO_2_ concentration (in ug/m^3^), AUE	27.7	1.9	27.7	1.9	27.7	1.9	0.922
NO_2_ concentration (in ug/m^3^), TOE	− 1.6	0.6	− 1.6	0.6	− 1.6	0.7	0.662
Damp stains in the house (% of the time yes), AUE	22	34	22	34	26	36	0.014
Damp stains in the house (% from no to yes), TOE	10	295	10	246	11	49	0.553
**Biological exposures**							
Body mass index (in kg/m^2^), AUE	25.6	3.5	25.4	3.3	26.9	4.2	< 0.001
Body mass index (in kg/m^2^), TOE	0.7	0.7	0.6	0.7	0.8	0.9	< 0.001
Use of high blood pressure medication (% of the time yes), AUE	10	21	9	20	15	25	< 0.001
Use of high blood pressure medication (% from no to yes), TOE	18	609	16	462	27	147	< 0.001

The AUE and TOE indicate the average value of the exposure over time and the average trend in the exposure over time for continuous exposures, respectively. For the categorical exposures, the proportion of the time that participants were in a particular category (AUE) and the proportion of participants for whom a change from one reference category to another category occurred during the rounds (TOE) is presented.

*AUE* Area-Under-the-Exposure, *TOE* trend-of-the-exposure.

*P*-values were tested using the independent samples t test and chi-square test.

### Predictors of self-perceived health (Step 2–5)

We then used RF to analyze which longitudinal exposures had the greatest predictive value for self-perceived health. The AUC of the RF model including all 96 exposures and with the optimal parameter settings for predicting self-perceived health on the training dataset was 0.742 (Supplementary Text S3). Fitting this model on the test dataset resulted in a slightly lower AUC of 0.707 (95% confidence interval: 0.655–0.759) (*Step 2–3*) (Table 3). At the optimal threshold in the ROC curve, sensitivity was 0.593 and specificity was 0.725. The average estimated risk of poor self-perceived health given by RF in the test dataset was 17.4%, which is comparable to the observed prevalence of poor self-perceived health of 15.9%. This is in line with the calibration curve in Supplementary Fig. S2, which indicates that the predicted risks of poor self-perceived health broadly correspond to the observed proportions, with the curve being generally close to the diagonal.

**Table 3:** Prediction performance metrics for the total model and the models without a particular domain of exposures.

Model	Optimal threshold ROC curve					Sensitivity and specificity at a predefined point of 0.5	
AUC (95% CI)	Threshold	Specificity	Sensitivity	Sensitivity + specificity	Accuracy	Specificity	Sensitivity
**Total**							
0.707 (0.655–0.759)	0.789	0.725	0.593	1.318	0.704	0.777	0.787
**Without demographic exposures**							
0.684 (0.630–0.739)	0.792	0.713	0.565	1.278	0.690	0.767	0.759
**Without lifestyle exposures**							
0.695 (0.642–0.747)	0.875	0.494	0.815	1.309	0.545	0.774	0.806
**Without environmental exposures**							
0.702 (0.650–0.754)	0.866	0.539	0.796	1.335	0.580	0.774	0.796
**Without biological exposures**							
0.669 (0.611–0726)	0.811	0.645	0.611	1.256	0.640	0.730	0.685

**Figure 4:**
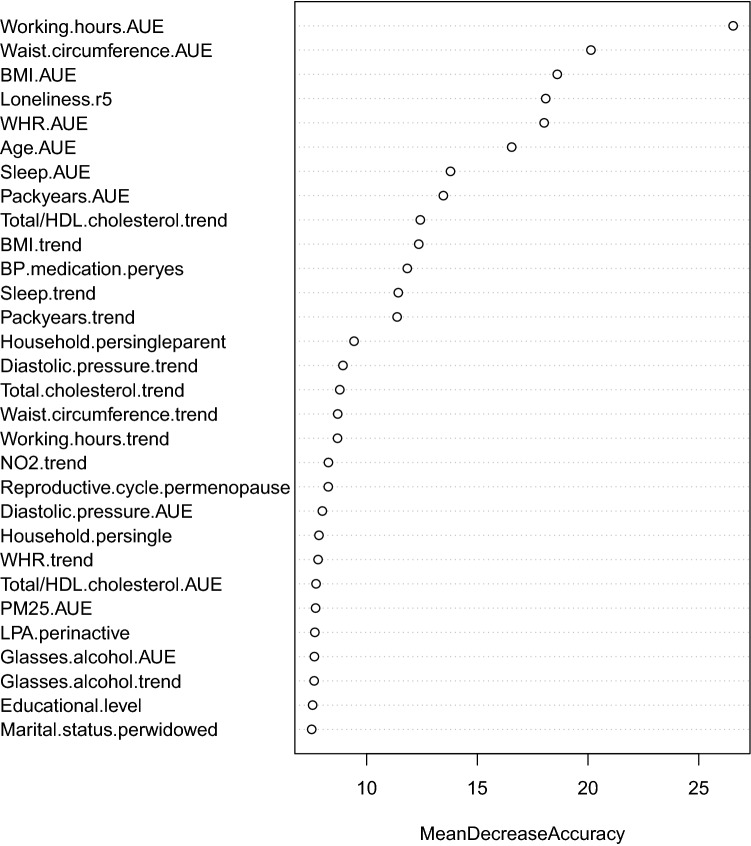
Variable importance ranking of the 30 most important exposures in predicting self-perceived health. The x-axis displays the mean decrease in accuracy that occurs when a particular exposure is permuted randomly in the random forest. AUE, Area-Under-the-Exposure; BMI, body mass index; BP, blood pressure; LPA, leisure time physical activity; r5, round 5; trend, Trend-of-the-Exposure; WHR, waist/hip ratio.

**Figure 5:**
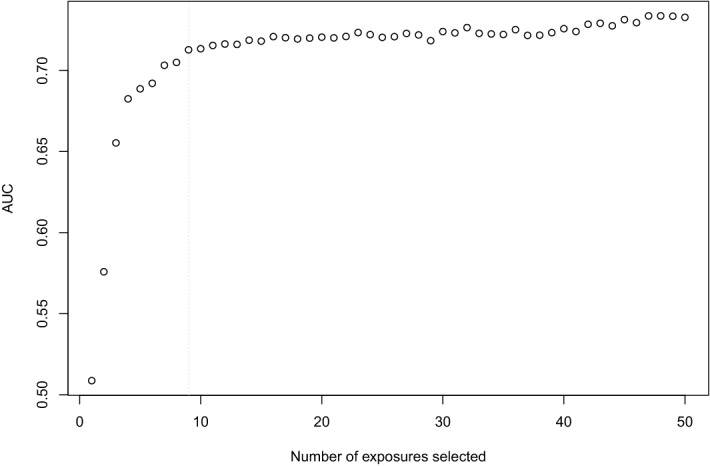
Exposure selection through cross-validation showing the prediction performance (Area-Under-the-Curve, AUC) (y-axis) of the model using a particular number of top-ranked exposures (x-axis). The dotted gray line represents the optimum number of exposures to select (q = 9).

Figure 4 displays the top 30 most important exposures in predicting self-perceived health based on the RF model (*Step 4*) performed on the entire dataset. To determine the number of top-ranked exposures needed to obtain an equally good prediction performance as in the model with all 96 exposures, we applied cross-validation on the training dataset (*Step 5*) (Fig. 5). The prediction performance sharply increased when selecting the first four exposures (AUC = 0.682). The AUC further increased when selecting between 5 and 9 exposures (AUC = 0.713), after which the curve flattened. Therefore, the optimum number of exposures to select was set at 9 exposures (Table 4). Applying the model with 9 exposures on the test dataset resulted in an AUC of 0.679 (95% CI 0.625–0.733, sensitivity = 0.685 and specificity = 0.595 at optimal threshold in the ROC curve), which was slightly lower than the AUC of 0.707 in the model with all 96 exposures.

**Table 4:** Top 9 predictors of self-perceived health.

**#**	Exposure	Label	Type	Round	Domain
1	Working hours	In hours per week	Average over time	r2–r5	Demographic
2	Waist circumference	In centimeters	Average over time	r2–r5	Biological
3	Body mass index	In kg/m^2^	Average over time	r1–r5	Biological
4	Loneliness	On a scale from 0 to 11	Measured in round 5	r5	Environmental
5	Waist/hip ratio	Ratio	Average over time	r2–r5	Biological
6	Age	In years	Average over time	r1–r5	Demographic
7	Sleep duration	In hours per day	Average over time	r1–r5	Lifestyle
8	Smoking pack years	In pack years	Average over time	r1–r5	Lifestyle
9	Total/HDL cholesterol ratio	Ratio	Average trend over time	r1–r5	Biological

### Relation between predictor and self-perceived health (Step 6)

**Figure 6:**
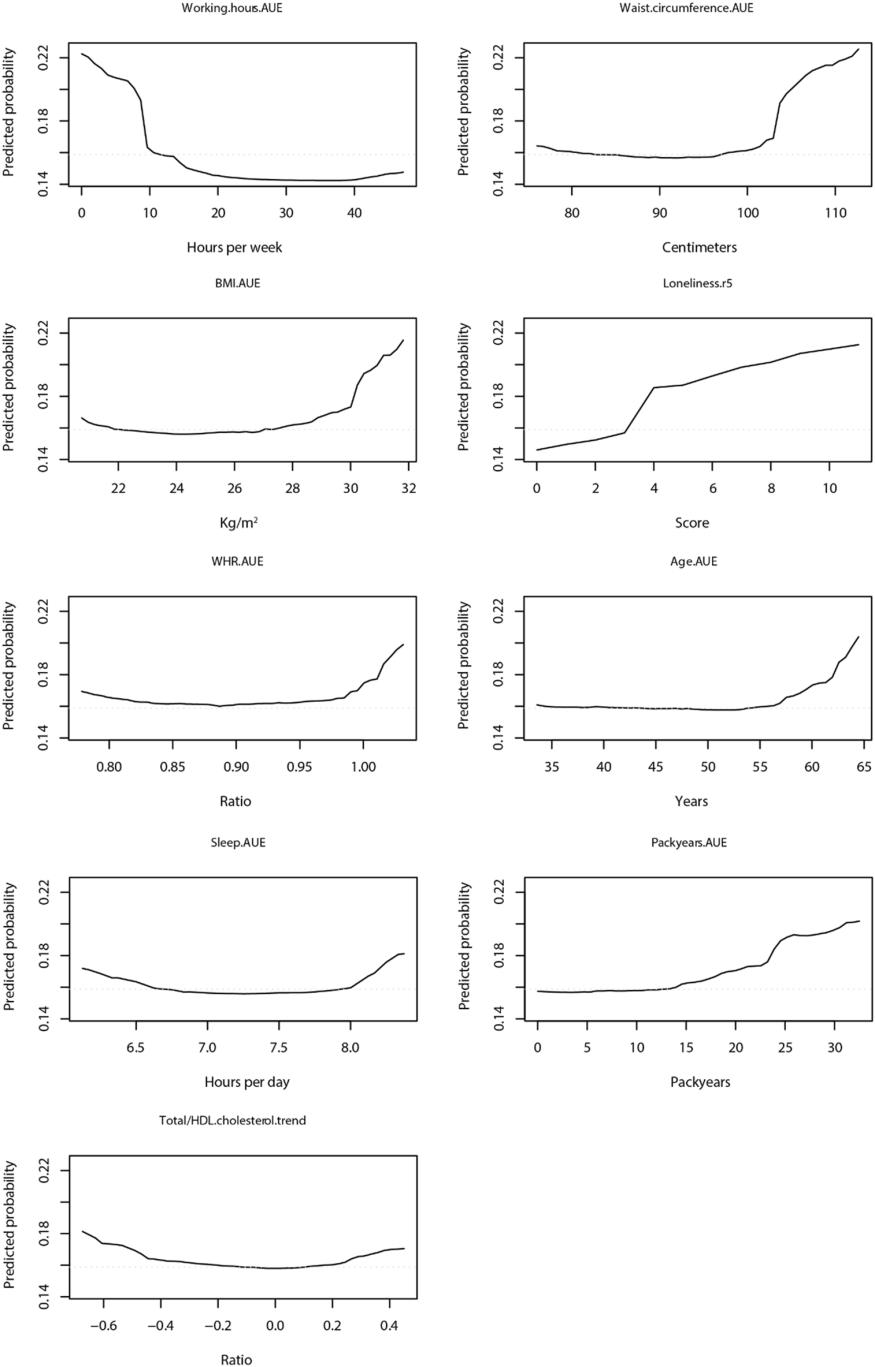
Partial dependence plots (PDPs) of the relation between predictors of self-perceived health and poor self-perceived health. The dotted gray line represents the reference value, i.e. the predicted outcome corresponds to the prevalence of poor perceived health in the total population at round 6 (0.16). AUE, Area-Under-the-Exposure; BMI, body mass index; r5, round 5; WHR, waist/hip ratio.

We then plotted the relation between the top 9 predictors and poor self-perceived health in PDPs (Fig. 6) and ALE plots (Supplementary Fig. S3) (*Step 6*). To illustrate, having worked on average < 10 h/week over time was predictive of poor perceived health. An advantage of these plots is that they facilitate automatic interpretation of non-linear relations. To avoid presenting results based on a small number of observations, we only plotted values from the 5^th^-95^th^ percentile of the predictor on the x-axes. Similar slopes were observed when using ALE plots (Supplementary Fig. S3), as the sign of the slopes (positive/negative) corresponds with the slopes in the PDPs. Supplementary Fig. S4 presents the distribution of the values of the predictors.

## Discussion

In this study, we described an approach based on ML to identify the exposures that predict self-perceived health best in a 30-year cohort study. Our approach involves (1) preprocessing the repeated measurements of exposures by constructing measures for the average value and trend over time of the exposures, (2) applying RF to build and optimize the prediction model, and using the AUC to determine the corresponding prediction performance, (3) ranking the exposures according to their contribution to the prediction performance, (4) selecting the exposures that all together more or less determine the overall prediction performance, and (5) using PDPs and ALE plots to determine the nature of their relation with the outcomes.

Our approach revolves around several key principles. First and foremost, a non-parametric approach seems well suited to an exploratory study. From the perspective of a statistician, data are generated by some stochastic model $y = f\left( x \right)$. In contrast to traditional regression approaches, ML approaches often make very few assumptions on the functional form of $f\left( x \right)$^5^. (One exception would, for instance, be LASSO^4^). The goal of many exposome studies is to explore associations between exposure and outcome, when there typically exists little to no a priori knowledge on how each exposure is related to the outcome, or on their relative importance. For these studies there is not necessarily a strong reason to assume any specific functional form, especially when the data are high dimensional. Such assumptions could comprise the number of exposures to include, the linearity of relations, and the absence of interaction effects. Assuming a wrong functional form may even lead to wrong conclusions in some cases^6^. For instance, if a linear relation between exposure and outcome is imposed on what is actually a parabolic relation, the corresponding regression parameter estimate is not informative, and could lead to not identifying this exposure as a relevant predictor. In our application we found that most exposures had non-linear relations with the outcome, which suggests that the risk of wrongly imposing a linear relationship is not negligible.

Second, it is difficult for any researcher to perform model and variable selection in practice, especially for high dimensional data. Even for our setting (96 exposures), there is a risk of overfitting^4^. Severe overfitting not only casts doubts on the prediction model, but also on the predictors it indirectly inferred while training. ML approaches automate model selection by finding a functional form that maximizes prediction accuracy, while using strategies (based on cross-validation and related techniques) to assess out-of-sample error and minimize the risk of overfitting. By contrast, stepwise selection methods completely neglect out-of-sample error and are thus prone to overfitting^28^, yet are amongst the most popular variable selection methods in epidemiology^29^. Furthermore, these methods completely neglect multiple testing issues, which is especially a problem in high dimensional settings^30^.

Third, a combination of data pre-processing and post-hoc visualization techniques can generally be used to make ML models more interpretable in longitudinal exposome studies. Since individual exposure can change over time, the trajectory of exposure may be predictive. Therefore, to facilitate interpretation, we created aggregations of repeated exposure measurements, as has been recommended previously^12^. In our study we represented the trajectories by considering both the average exposure over time and the average trend in the exposure, that describe the persistence and evolution of exposure respectively. These representation measures can then be used in the ML model. After training the ML model, visualization techniques such as PDPs^31^ and ALE plots^25^ can help in interpreting the ML model. For any given exposure, these plots illustrate how the prediction of the outcome changes on average when changing the values of that exposure while keeping all other exposures constant at their original values. Although it is not possible to produce straightforward regression coefficients, such plots can always be applied to obtain an interpretation that is similar, in terms of the sign and magnitude of the effect size.

In the current study, all investigated domains (demographic, lifestyle, environmental, and biological exposures) were represented in the identified predictors of self-perceived health. This agrees with prior prediction and risk assessment studies with health outcomes such as self-perceived health, mortality, and disability-adjusted life-years that also identified exposures from different domains to be important in predicting these health outcomes^32–34^. While the biological factors were relatively overrepresented in the top-ranked predictors, this exposure domain did not outperform the other domains in its relative contribution to predicting self-perceived health (Table 3). Therefore, it cannot be concluded that self-perceived is primarily predicted by a particular domain. Instead, applying a broad range of exposures across domains (i.e. an exposome framework) seems to be more appropriate in this context. To this end, the approach applied in the current study is helpful, because it provides a direct comparison and ranking of the predictive performances of different types of predictors for self-perceived health.

Across domains, the average number of working hours over time was by far the leading predictor of self-perceived health at older age. Having on average no working hours over time was in particular predictive of having poor perceived health (Fig. 6). In correspondence, in earlier studies into the predictive value of exposures across different domains on health outcomes, having a history of unemployment was among the top 5 factors associated with the greatest risk of poor health and mortality^33,35^.

This paper is intended to provide other researchers with an example and tutorial of how ML can act as an useful addition to an epidemiologist’s toolkit. It can thus provide other researchers with an application of how to use an ML algorithm to answer a public health research question. However, the proposed approach only covers the bare necessities and should therefore be seen as a point of departure for epidemiologists. Limitations of our approach include the following. First, our approach was illustrated using RF, but many algorithms exist. As the focus of many epidemiologist and public health researchers is on the application itself and the relevance for health policy, only one algorithm was included in this paper and RF was considered a good choice for this purpose. However, some other algorithms that can be considered are other tree-based methods (e.g.^36^), support vector machines, and neural networks^7,14^. In addition, we used the AUC of the ROC curve to assess the discriminative quality of our model, but alternative measures for discrimination are available too (e.g. the scaled Brier score)^37^.

Second, alternative strategies may exist to select the most important variables. Our strategy is based on considering the number of exposures as a tuning parameter using cross-validation and visually inspecting the exposures that substantially contribute to the prediction performance. There is room for interpretation differences here. Furthermore, the interpretation is strengthened by the modest contribution by many exposome variables. Such exposures may in truth be associated, but based on a prediction performance based metric they tend to be not as easily identified. It may therefore be more worthwhile to look at alternative variable selection strategies^38,39^, or the use of p-values in variable importance^40,41^. Furthermore, strongly correlated exposures may be more difficult to interpret in variable importance rankings, and may require other approaches to improve interpretation^42^.

Third, our approach does not take into account potential informative censoring and/or missingness in longitudinal studies. The dropout of individuals may be related to their characteristics, and some approaches have been developed to deal with this^43,44^.

Fourth, our approach has not taken into account class imbalance in the outcome. When the dataset is highly imbalanced, i.e. one class of the outcome is strongly overrepresented compared to another class, the ML algorithm will mainly focus on predicting the majority class well, whereas the minority class is most likely to be the class of interest^45^. Class imbalance in our case study was limited, but in cases of severe imbalance (e.g. where one class of the outcome for example includes 1% and the other 99% of the cases), it may be worthwhile to apply a balancing technique such as over-sampling or under-sampling^45,46^.

Finally, it is important to note that the proposed approach focuses on prediction of a health outcome and it does not aim to estimate causal effects. Although there has been less emphasis in the literature on using ML for causal inference, this is currently a highly emerging field of research^9^. Some interesting new developments include for example causal forests and causal structure learning^47,48^.

## Conclusion

We proposed an approach to predict health outcomes based on longitudinal exposures and to identify relevant predictors. This approach combines the use of ML, with its many attractive properties, with visualization methods to facilitate interpretability. We show that ML can be interpreted more than is widely believed, and can be a valuable asset in epidemiological research. With this paper, we aim to support others in implementing ML techniques for studying (longitudinal) predictors of health outcomes.

## Supplementary Information

Supplementary Information.

## Data Availability

The datasets generated during and analysed during the current study are not publicly available due to ethical restrictions related to participant consent but are available from the corresponding author on reasonable request.
