# Supplementary material for: Using random forest to identify longitudinal predictors of health in a 30-year cohort study
Source: Sci Rep. 2022 Jun 20;12:10372. doi: 10.1038/s41598-022-14632-w (PMC9209521; doi:10.1038/s41598-022-14632-w)
Supplement: Supplementary file 1 — Supplementary Information. [file 41598_2022_14632_MOESM1_ESM.pdf]

SUPPLEMENTARY INFORMATION

Using random forest to identify longitudinal predictors of health in a 30-year cohort study

Bette Loef, Albert Wong, Nicole A.H. Janssen, Maciek Strak, Jurriaan Hoekstra, H. Susan J. Picavet, H.C. Hendriek Boshuizen, W.M. Monique Verschuren, Gerrie-Cor M. Herber

Supplementary Text S1. Statistical analysis

- **Supplementary Figure S1.** Hypothetical example of the Area-Under-the-Exposure (AUE) and Trend-of-the-Exposure (TOE) for the continuous exposure body mass index (BMI), showing a low AUE and increasing TOE (a), a low AUE and decreasing TOE (b), a high AUE and increasing TOE (c), and a high AUE and decreasing TOE (d). A missing point at a certain measurement round indicates a missing value.
- **Supplementary Table S1.** Hypothetical example of the Area-Under-the-Exposure (AUE) and Trend-of-the-Exposure (TOE) for the categorical exposure smoking status

Supplementary Text S2. Statistical code

Supplementary Table S2. Characteristics of the study population stratified by good or poor perceived health status

Supplementary Text S3. Prediction performance of the RF model

- **Supplementary Table S3.** Prediction performance metrics for the total model and the models without a particular domain of exposures
- **Supplementary Figure S2.** Calibration curve showing the predicted probabilities and the observed outcome (i.e. poor self-perceived health) percentages in the test dataset. The dotted line represents perfect calibration. None of the participants had a predicted probability of 60% or higher, and only one participant was included with a predicted probability >55%, explaining the spike at the end of the line.

Supplementary Figure S3. Accumulated local effects plots of the relation between predictors of self-perceived health and poor self-perceived health. The dotted gray line represents the reference value, i.e. the local effect is 0. AUE, Area-Under-the-Exposure; BMI, body mass index; r5, round 5; WHR, waist/hip ratio.

Supplementary Figure S4. Distribution of the values of the predictors of self-perceived health. AUE, Area-Under-the-Exposure; BMI, body mass index; r5, round 5; WHR, waist/hip ratio.

Supplementary Text S1. Statistical analysis

In the next section, we describe the different steps towards a prediction model for self-perceived health based on demographic, lifestyle, environmental, and biological exposures using RF. We begin by explaining how longitudinal exposures were assessed (Step 1), followed by a description of the RF algorithm (Step 2), how it was optimized using tuning parameters, and how its prediction performance was determined (Step 3). This is followed by an explanation of how the importance of the individual exposures in predicting health was assessed (Step 4) and what exposures needed to be included to create a parsimonious model with good performance using exposure selection through cross-validation (Step 5). Lastly, the visual representation of the relation between the most important exposures and health using partial dependence and accumulated local effects plots is explained (Step 6).

Step 1: Assessing longitudinal exposures

Most exposures were assessed during multiple measurement rounds. Using the exposure variable set as-is implies that each exposure at a given round is considered a potential predictor. To facilitate interpretation, we pre-processed and summarized continuous exposures, by introducing the so-called Area-Under-the-Exposure (AUE) and the Trend-of-the-Exposure (TOE).

The AUE represents the *average* of the continuous exposure during the rounds leading up to the round at which the self-rated health outcome is observed. Underlying this construct is the assumption that prolonged exposure over time is in particular predictive of the outcome (self-perceived health). The AUE is computed for each exposure variable and for each individual separately, by plotting observed exposure values against rounds, connecting the values with lines, and determining the *average* area under these lines (that is, the total area under the lines divided by the number of exposure measurement rounds minus one). The higher the AUE, the higher the prolonged exposure over the life course.

The TOE represents the *average trend* in the exposure. Here the assumption is that a positive or negative trend is also predictive of the outcome. The TOE is computed for each individual and exposure, through determining the slope in exposure for each pair of subsequent rounds (slope for round 1-2, for round 2-3, etc.), and taking the average over that. A positive value for TOE indicates an upward trend in exposure, whereas a negative value indicates a downward trend.

For categorical exposure variables the AUE and TOE were defined differently. The AUE was defined as the proportion of rounds that the individual occupied a certain state (which corresponds to a unique category of the variable). For instance, for the smoking variable the AUE was defined as the proportion of time that the individual was a smoker. The TOE was defined as an indicator variable, that signified whether a change from one reference category to another category had occurred during the rounds. For instance, whether an individual had gone from 'no smoking' to 'smoking'. For every categorical exposure, one TOE variable indicating a change from a reference category to another category was included.

Supplementary Figure S1 and Supplementary Table S1 provide an example of the calculation of the AUE and TOE for a continuous exposure (BMI) and a categorical exposure (smoking status), respectively.

An advantage of this approach is that the AUE and TOE can also be calculated in case of missing values, which is a common problem in longitudinal cohort studies. To calculate the longitudinal exposures, participants had to have a value for the exposure in at least two rounds. Participants with missing values for an exposure on 4 or 5 rounds were labelled as missing (i.e. 99999, this is an outlier value for continuous exposures and a missing category for categorical exposures) on that particular longitudinal exposure. In total, 75% of the participants had no missing values on the longitudinal

exposures, 7% of the participants had a missing value on one longitudinal exposure, 5% on two longitudinal exposures, and 13% on three or more longitudinal exposures.

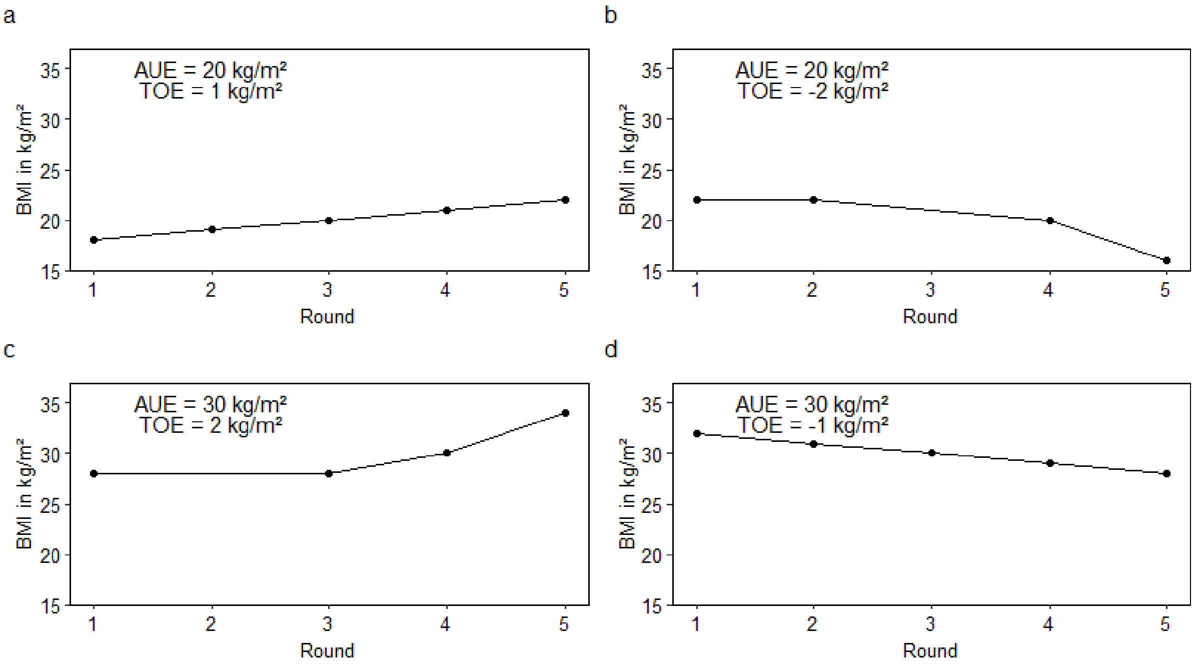

Supplementary Figure S1. Hypothetical example in different individuals of the Area-Under-the-Exposure (AUE) and Trend-of-the-Exposure (TOE) for the continuous exposure body mass index (BMI), showing a low AUE and increasing TOE (a), a low AUE and decreasing TOE (b), a high AUE and increasing TOE (c), and a high AUE and decreasing TOE (d). A missing point at a certain round indicates a missing value.

Supplementary Table S1. Hypothetical example of the Area-Under-the-Exposure (AUE) and Trend-of-the-Exposure (TOE) for the categorical exposure smoking status

	Round 1	Round 2	Round 3	Round 4	Round 5	Proportion of the time being a smoker (AUE)	Change from never smoker to smoker (TOE)
a	Never	Never	Smoker	Smoker	Former	40%	Yes
b	Smoker	Former	-	Former	Former	25%	No
c	Never	-	-	Never	Never	0%	No

-, missing value

In our approach we included measures that represent the average value and average trend of an exposure measured during multiple rounds. If considered appropriate, other summary measures could also be included, for example a measure of the standard deviation of the AUE and TOE to include variation in these measures. Furthermore, in our approach the AUE and TOE were based on exposures measured in round 1 through round 5 while the outcome measure was assessed at round

6. However, one could also consider including exposures assessed at round 6 in the summary measures.

Step 2: Choosing a ML algorithm: random forest

To analyze what longitudinal exposures had the greatest predictive value for self-perceived health, the random forest (RF) algorithm was used [1]. This non-parametric machine learning algorithm is one of the top-performing algorithms in classification problems [2] and consists of an ensemble of decision trees that predict the outcome measure. In our study, decision trees can be used to classify participants as having a good vs. poor perceived health status based on the values of the longitudinal exposures. Decision trees are an easy method to interpret, but they are prone to overfitting and thus yield predictions that are not easily generalizable to datasets other than the dataset on which they were built. RF deals with this limitation by building a forest of decision trees [1]. To this end, RF first creates a bootstrap sample by randomly selecting individuals, with replacement, from the original dataset. The bootstrap sample consists of the same number of observations as the original dataset. On each bootstrap sample a decision tree is built by repeatedly making binary splits (i.e. dividing the data into two partitions). Each split is made by first taking a random subset of exposures, then picking one exposure and an accompanying split point such that a pre-specified criterion is optimized. For RF, the Gini Impurity measure is the default criterion to measure how well the split is able to distinguish good from poor health [1]. Splits are made until a certain tree depth is reached, or when a partition has reached a minimum node size (see Step 3). The final partitions are called terminal nodes. A prediction for a new observation can be made by identifying the partition in which the new observation falls, and then by assigning the majority vote in the partition to the new observation. Within RF, the step of creating a bootstrapped dataset and building a decision tree on it is repeated many times, resulting in a forest of trees. The entire forest is used to obtain predicted probabilities of poor health. For every individual, the predicted class (i.e. good or poor health) by

each decision tree is obtained, and then the proportion of trees that predict poor health is used as the predicted probability of poor health.

RF typically measures the prediction performance through the so-called out-of-bag (OOB) error [1]. A limitation of using the OOB error as prediction performance metric is that it is directly tied to accuracy (number of individuals whose class is predicted correctly) and can be misleading when the outcome measure in the dataset is imbalanced. In our study, nearly 16% of individuals had poor perceived health. A ‘dummy’ (and naïve) classifier would classify all individuals as having good health, leading to an error rate of 16%. If the RF achieves an OOB of 16%, it would seem that it performs quite reasonably, when it actually does no better than the dummy classifier. To overcome this issue, we made use of the Receiver Operating Characteristic (ROC) curve and its Area-Under-the-Curve (AUC) instead. The AUC is not dependent on the prior class probabilities. Next to the AUC, we reported the sensitivity, specificity, and accuracy belonging to (a) the optimal threshold that we defined as the threshold in the ROC curve for which the sum of sensitivity and specificity is maximized, and (b) a predefined threshold of 0.5.

To examine the extent to which the observed outcomes and predicted probabilities were in agreement, the average predicted risk was compared with the overall outcome rate (i.e. calibration-in-the-large) [3]. Furthermore, a calibration curve was constructed plotting the predicted probabilities against the percentage of observed outcomes.

Step 3: Optimizing prediction performance

In general, the prediction performance of the RF algorithm is adequate when using the default setting for its tuning parameters [4]. However, these parameters can be tuned to further improve prediction performance. Commonly used tuning parameters are *mtry*, *ntrees*, *nodesize* and *maxnodes* [4,5]. The random subset of exposures that is used at each split of the tree can be altered,

this is called mtry. The default of mtry for classification (i.e. categorical outcome) is the square root of the number of included exposures. The number of trees (ntrees) of which the RF consists should be set sufficiently high (default = 500). Nodesize, the minimum number of observations in the final nodes (the leaves of the tree), is usually set at 1 for classification and at 5 for regression (i.e. continuous outcome). By adjusting maxnodes, which is the maximum number of terminal nodes that trees in the forest can have, the depth of the trees can be controlled. By default, trees are grown to the maximum possible.

In order to choose the optimal parameter settings, we randomly divided the dataset in a training dataset and a test dataset using a 80%/20% split with a similar distribution of the proportions of good/poor perceived health in both datasets. Next, we selected the combination of settings for the tuning parameters that produced the highest prediction performance on the training dataset with a grid search in combination with 5-fold cross-validation (the choice of k in k-fold cross-validation is usually 5 or 10) with R-package caret [6]. Lastly, the model with the optimal parameter settings was used to make predictions on the test dataset and the corresponding ROC curve and AUC were determined.

Step 4: Ranking the variable importance

One of the primary outcomes of RF is the variable importance ranking, which reflects a ranking of the importance of the exposures in the prediction performance of the RF. For classification, the variable importance ranking plot shows a list of ‘most relevant’ variables, that are ranked by mean decrease in accuracy (MDA) that occurs when the particular exposure is permuted randomly in the RF. If the exposure is strongly predictive of the outcome, the random permutation will lead to a large MDA. As the MDA indicates how much accuracy the prediction model losses by removing each exposure, it provides insight into the additive predictive value of a particular exposure in addition to all other exposures. Variables with a large MDA can be considered as strong independent predictors

of the outcome. The variable importance ranking can thus be used to investigate and identify associations between exposures and the outcome. Besides the effect size of the exposure, the RF variable importance ranking also automatically captures non-linear and interaction effects without hard-coded specification of these effects [4]. We obtained the variable importance ranking by taking the optimal tuning parameter settings and fitting a RF on the entire dataset, analogous to what would be done in typical epidemiological/statistical association studies. In the current study, we show the 30 top-ranked exposures in the variable importance.

Step 5: Selecting exposures through cross-validation

A good prediction model is characterized by its ability to strike a balance between prediction accuracy and parsimony, which means that exposures that do not or hardly contribute to the prediction performance of the model should be excluded. In other words, the parsimonious model would be at least not (substantially) worse than a model with all variables. The parsimonious model cannot be obtained through the variable importance ranking (alone), but must be obtained by considering the number of variables included in the final prediction model as a tuning parameter (*q*), and evaluating the relation between q and the prediction performance through a separate procedure. This involves (a) creating 5 partitions in the 80% training dataset, (b) forming a temporary training dataset based on 4 partitions and a temporary validation dataset based on 1 partition, and training a RF, (c) choosing a value of q, and selecting the top-ranked q variables from the variable importance ranking from the trained RF, (d) building a new model on the training dataset with just the q variables, and (e) making predictions with this new model on the validation dataset. This procedure is repeated until all partitions have been used as a validation dataset, and until all q values have been used. Afterwards, the AUC was estimated for each choice of q, and plotted against each other. The optimal value for q was chosen based on the flattening of the resulting curve. Next, the optimal value for q was used to make predictions on the 20% test dataset and to determine the corresponding ROC curve and AUC.

Step 6: Plotting partial dependence plots and accumulated local effects plots

The variable importance ranking identifies the most important exposures that predict self-perceived health. However, it does not provide information about the shape of the relation between the exposure and self-perceived health. To visualize this relation, partial dependence plots (PDP) [7] and accumulated local effects (ALE) plots were produced [8]. PDP plots the value of the average predicted outcome on the y-axis against each value of the exposure on the x-axis. The average predicted outcome for a given exposure value was obtained by setting the exposure in the sample population to that value and leaving all other exposures unchanged, and then to generate predictions, and average over them. On the other hand, ALE plots only look at the local effects of an exposure, i.e. the effect is estimated in a subpopulation that is located within a certain range of the exposure. An advantage of the ALE plots is that they largely avoid extrapolation of the effect at values of the exposure that do not occur in (combination with certain values of another exposure in) the dataset, which is especially a problem when there are highly correlated exposures [8]. However, a consequence of this is that the local effects are only applicable to the specific subpopulation for which it was calculated, and therefore it is difficult to interpret and compare the size of different local effects. In the current study, both PDPs and ALE plots were plotted for the number of most important exposures selected through cross-validation as described above. The PDPs provide a general sense of the effect size, while the ALE plots were used to check whether the slopes as observed in PDPs are possibly the result of extrapolation issues.

Analyzes were performed using R Version 4.0.2. (http://www.R-project.org/). RF analyses were conducted using the R-package randomForest [9]. The R-package caret was used to tune the parameters of the RF analyses [6]. The R-package iml was used to plot the ALE plots [10].

REFERENCES

1 Breiman, L. Random forests. *Machine learning* **45**, 5-32 (2001).

2 Fernández-Delgado, M., Cernadas, E., Barro, S. & Amorim, D. Do we need hundreds of classifiers to solve real world classification problems? *J Mach Learn Res* **15**, 3133-3181 (2014).

3 Van Calster, B., McLernon, D. J., van Smeden, M., Wynants, L. & Steyerberg, E. W. Calibration: the Achilles heel of predictive analytics. *BMC Med* **17**, 230 (2019).

4 Probst, P., Wright, M. N. & Boulesteix, A. L. Hyperparameters and tuning strategies for random forest. *Wiley Interdiscip Rev Data Min Knowl Discov* **9**, e1301 (2019).

5 Scornet, E. Tuning parameters in random forests. *ESAIM: Proceedings and Surveys* **60**, 144-162 (2017).

6 Kuhn, M. Building predictive models in R using the caret package. *Journal of statistical software* **28**, 1-26 (2008).

7 Hastie, T., Tibshirani, R. & Friedman, J. The elements of statistical learning. Second ed: New York: Springer (2009).

8 Apley, D. W. & Zhu, J. Visualizing the effects of predictor variables in black box supervised learning models. *J R Stat Soc Series B* **82**, 1059-1086 (2020).

9 Liaw, A. & Wiener, M. Classification and regression by randomForest. *R news* **2**, 18-22 (2002).

10 Molnar, C., Casalicchio, G. & Bischl, B. iml: An R package for interpretable machine learning. *J Open Source Softw* **3**, 786 (2018).

Supplementary Text S2. Statistical code

```
##Using random forest to identify longitudinal predictors of health in a 30-
year cohort study
#Bette Loef, Albert Wong, Nicole A.H. Janssen, Maciek Strak, Jurriaan
Hoekstra, H. Susan J. Picavet, H.C. Hendriek Boshuizen, W.M. Monique
Verschuren, Gerrie-Cor M. Herber

##Legend:
#dataset.total: dataset including outcome measure and 96 exposures of 3419
participants
#y: outcome measure self-perceived health


##Step 1: Assessing longitudinal exposures

#Formula to calculate AUE and TOE
AUC.indicator <- function(data.xy){
  x.and.y <- na.omit(data.xy)
  n <- nrow(x.and.y)
  if (nrow(x.and.y)%in%c(0,1))
  {
    AUC <- NA
  } else {
    AUC <- sum((x.and.y[-1,1]-x.and.y[-n,1])*(x.and.y[-1,2]+x.and.y[-
n,2])/2)/(x.and.y[n,1]-x.and.y[1,1])
  }
  return(AUC)
}

trend.indicator <- function(data.xy){
  x.and.y <- na.omit(data.xy)
  n <- nrow(x.and.y)
  if (nrow(x.and.y)%in%c(0,1))
  {
    trend.indicator <- NA
  } else {
    trend.indicator <- mean((x.and.y[-1,2]-x.and.y[-n,2])/(x.and.y[-1,1]-
x.and.y[-n,1]))
  }
  return(trend.indicator)
}

#Example 1 caculating AUE en TOE
x <- c(1,2,3,4)
y <- c(3,2,2,1)
data.xy <- cbind(x,y);
plot(x=na.omit(data.xy)[,1],y=na.omit(data.xy)[,2],type="o", ylim=c(0,max(y,
na.rm=TRUE)))
```

```
AUC.indicator(data.xy)
trend.indicator(data.xy)

#Example 2 caculating AUE en TOE
x <- c(1,2,NA,4)
y <- c(3,2,NA,1)
data.xy <- cbind(x,y);
plot(x=na.omit(data.xy)[,1],y=na.omit(data.xy)[,2],type="o", ylim=c(0,max(y,
na.rm=TRUE)))
AUC.indicator(data.xy)
trend.indicator(data.xy)

#Specify longitudinal exposures x
x.names <- c('x1','x2','x3')

#Make formula for outcome measure y as a function of exposures x
formula.tmp <- as.formula(paste0("y~",paste(x.names,collapse="+")))


##Step 2: Choosing a ML algorithm: random forest + Step 3: Optimizing
prediction performance
library(caret)
library(mlbench)
library(pROC)
library(dplyr)

#Create training dataset and test dataset with a similar distribution of the
proportions of good/poor perceived health
set.seed(12345)
indexes <- createDataPartition(dataset.total$y,
  times = 1,
  p = 0.8,
  list = FALSE)
dataset.train <- dataset.total[indexes,]
dataset.test <- dataset.total[-indexes,]

#Create predefined folds
set.seed(12345)
cv_folds <- createFolds(dataset.train$y, k = 5, returnTrain = TRUE)

#Create tune control
tuneGrid <- expand.grid(.mtry = c(5:25))

ctrl <- trainControl(method = "repeatedcv",
  number = 5,
  repeats= 3,
  search = 'grid',
  classProbs = TRUE,
  savePredictions = "final",
```

```
  index = cv_folds,
  summaryFunction = twoClassSummary)

#Define other parameters
ntrees <- c(500, 1000)
nodesize <- c(1:10, 15, 20)

params <- expand.grid(ntrees = ntrees, nodesize = nodesize)

#Grid search
store_gridsearch <- vector("list", nrow(params))
for(i in 1:nrow(params)){
  nodesize <- params[i,2]
  ntree <- params[i,1]
  set.seed(12345)
  rf_model <- train(formula.tmp,
    data=dataset.train,
    method = "rf",
    metric = "ROC",
    tuneGrid = tuneGrid,
    trControl = ctrl,
    ntree = ntree,
    nodesize = nodesize
    )
  store_gridsearch[[i]] <- rf_model
}

#Combine results
results <- resamples(store_gridsearch)
summary(results)

#Choose optimal value for parameters based on grid search
mtry.opt      <- 10
ntree.opt     <- 1000
nodesize.opt  <- 7

#Test hold-out metrics
tuneGrid <- expand.grid(.mtry = mtry.opt)
set.seed(12345)
rf.fit <-  train(formula.tmp,
  data=dataset.train,
  method = "rf",
  importance=TRUE,
  metric = "ROC",
  tuneGrid = tuneGrid,
  trControl = ctrl,
  ntree = ntree.opt,
  nodesize = nodesize.opt)
```

```
caret::confusionMatrix(data = predict(rf.fit, dataset.train), reference =
dataset.train$y)
pred.prob <- predict(rf.fit, dataset.test, type = "prob")
result.roc <- roc(dataset.test$y, pred.prob$X1, auc = TRUE, ci = TRUE)
result.roc

par(pty = "s")
plot(result.roc)


##Step 4: Ranking the variable importance

#Fit Random Forest to total dataset with optimal parameter settings
set.seed(12345)
rf.total <- randomForest(
  formula = formula.tmp,
  data = dataset.total,
  ntree = ntree.opt, mtry = mtry.opt, nodesize = nodesize.opt,
  importance = TRUE)

# Plot variable importance
varImpPlot(rf.total, type = 1)


##Step 5: Selecting exposures through cross-validation

set.seed(12345)
indexes <- createDataPartition(dataset.total$y,
  times = 1,
  p = 0.8,
  list = FALSE)
dataset.80train <- dataset.total[indexes,]
dataset.20holdout <- dataset.total[-indexes,]

n.folds                 <- 5
n.selected.variables    <- 96
predictions.mat         <- matrix(NA, nrow=nrow(dataset.80train),
ncol=n.selected.variables)


set.seed(12345)
fold.indices            <- sample(x=rep(1:n.folds),
size=nrow(dataset.80train), replace=TRUE)

output.auc              <- data.frame(matrix(0,0,2))
names.output            <- c("n.selected","auc")
names(output.auc)       <- names.output
```

```
#Loop over each test-fold.
for (fold.tmp in 1:n.folds)
{
  trainingset <- dataset.80train[which(fold.indices!=fold.tmp),]
  testset     <- dataset.80train[which(fold.indices==fold.tmp),]

  set.seed(12345)
  #Use the optimal tuning parameters here (as found previously in step 3).
   rf.fit2 <- randomForest(
    formula = formula.tmp,
    data = trainingset,
    ntree = ntree.opt, mtry = mtry.opt, nodesize = nodesize.opt,
    importance = TRUE)
  importance.ordered <- importance(rf.fit2, type=1)[order(importance(rf.fit2, 
type=1)[,1], decreasing=TRUE),1]
  xlab.vec               <- names(importance.ordered)

  #Vary index for n.selected.variables. Train/Predict/Compute AUC each time.
  for (n.selected.tmp in 1:n.selected.variables)
  {
    if (n.selected.tmp%%5==0)
    {
      print(paste0("fold=", fold.tmp, "; n.selected=", n.selected.tmp, ": ", 
date()))
    }
    top.selected.tmp     <- names(importance.ordered[1:n.selected.tmp])
    formula.select.tmp   <- as.formula(paste0("y ~ ", 
paste0(top.selected.tmp, collapse=" + "))  )
    rf.tmp               <- randomForest(formula.select.tmp, data = 
trainingset, ntree = ntree.opt, mtry = mtry.opt, nodesize = nodesize.opt, 
importance = FALSE)
    predictions.mat[which(fold.indices==fold.tmp),n.selected.tmp]  <- 
predict(rf.tmp, newdata=testset, type="prob")[,2]
  }
}

for (n.selected.tmp in 1:n.selected.variables)
{
  test.tmp              <- predictions.mat[,n.selected.tmp]
  roc.fit               <- roc(as.numeric(as.character(dataset.80train$y)), 
test.tmp)
  row.tmp               <- c(n.selected.tmp, auc(roc.fit))
  output.auc.tmp        <- data.frame(matrix(row.tmp,1,2))
  names(output.auc.tmp)<- names.output
  output.auc            <- rbind(output.auc, output.auc.tmp)
}

head(predictions.mat,10)
head(output.auc,96)
```

```
##Step 6: Plotting partial dependence plots and accumulated local effects
plots

library(iml)

#Fit Random Forest to total dataset with optimal parameter settings
set.seed(12345)
rf.total <- randomForest(
  formula = formula.tmp,
  data = dataset.total,
  ntree = ntree.opt, mtry = mtry.opt, nodesize = nodesize.opt,
  importance = TRUE)

#PDP and ALE plots
pfun <- function(object, newdata) predict(object, newdata = newdata,
type="prob")[,2]
predictor <- Predictor$new(model = rf.total, data = dataset.total, y =
dataset.total$y, predict.fun = pfun)
cc <- "ratio"
n <- 5
imp <- FeatureImp$new(predictor, loss = "ce", n.repetitions = n, compare =
cc)

FeatureEffect$new(predictor, "x", method = "ale", center.at = NULL, grid.size
= 30)
```

Supplementary Table S2. Characteristics of the study population stratified by good or poor perceived health status

			Total population (n=3419)		Good perceived health (n=2876)		Poor perceived health (n=543)		
**Exposure**	**Label**	**Type**	*Mean/%*	*SD/n*	*Mean/%*	*SD/n*	*Mean/%*	*SD/n*	*p-value*
Demographic exposures									
Sex	female	%	52.94	1810	52.43	1508	55.62	302	0.188
Age	in years	AUE	48.70	9.29	48.22	9.09	51.22	9.91	<0.001
Age	in years	TOE	5.23	0.10	5.23	0.09	5.25	0.12	0.001
Educational level	higher vocational education or university	%	25.71	879	26.81	771	19.89	108	<0.001
Nationality	Dutch	%	99.01	3385	99.30	2856	97.42	529	<0.001
Marital status	married	% of the time	81.03	32.33	82.03	31.33	75.72	36.78	<0.001
Marital status	widow/widower	% of the time	2.85	13.12	2.54	12.18	4.54	17.20	0.001
Marital status	divorced	% of the time	5.83	18.86	5.31	17.87	8.59	23.22	<0.001
Marital status	from married to widowed or divorced	%	16.65	569	15.86	456	20.85	113	0.005
Household composition	with partner (and children)	% of the time	84.52	29.80	85.85	28.32	77.42	35.90	<0.001
Household composition	single-parent household	% of the time	4.17	13.64	3.95	12.78	5.30	17.47	0.037
Household composition	single household	% of the time	9.21	24.42	8.17	22.89	14.76	30.79	<0.001
Household composition	from with partner (and children) to single-parent or single household	%	14.25	482	14.18	404	14.63	78	0.836
Working hours	in hours per week	AUE	20.92	15.88	22.05	15.55	14.89	16.27	<0.001
Working hours	in hours per week	TOE	-1.18	7.51	-1.06	7.60	-1.79	6.98	0.040
Lifestyle exposures									
Alcohol use	no, I stopped using alcohol	% of the time	1.61	7.92	1.40	7.56	2.73	9.55	<0.001
Alcohol use	every now and then or yes	% of the time	89.35	25.63	90.44	24.28	83.55	31.23	<0.001
Alcohol use	from never user to current user	%	8.70	297	8.74	251	8.49	46	0.914
Number of glasses of alcohol	in glasses per day	AUE	1.08	1.24	1.09	1.22	1.01	1.35	0.174
Number of glasses of alcohol	in glasses per day	TOE	0.01	0.30	0.02	0.27	-0.03	0.41	<0.001
Smoking status	smoker	% of the time	22.76	35.74	21.37	34.77	30.10	39.76	<0.001
Smoking status	former smoker	% of the time	39.62	41.94	39.88	42.14	38.20	40.86	0.391
Smoking status	from never smoker to smoker	%	1.81	62	1.84	53	1.66	9	0.906
Number of cigarettes	in cigarettes per day	AUE	13.51	6.73	13.04	6.63	15.28	6.82	0.067
Number of cigarettes	in cigarettes per day	TOE	-0.13	3.33	-0.09	3.29	-0.27	3.47	0.526
Smoking pack years	in pack years	AUE	8.72	11.63	8.06	10.98	12.21	14.13	<0.001
Smoking pack years	in pack years	TOE	0.83	2.23	0.79	2.08	1.08	2.87	0.005
Occupational physical activity	sedentary job	% of the time	28.40	35.78	30.04	36.45	19.69	30.59	<0.001
Occupational physical activity	standing job	% of the time	22.38	26.56	23.12	27.24	18.42	22.17	<0.001

Exposure	Label	Type	Total population (n=3419)		Good perceived health (n=2876)		Poor perceived health (n=543)		
			Mean/%	*SD/n*	*Mean/%*	*SD/n*	*Mean/%*	*SD/n*	*p-value*
Occupational physical activity	(heavy) manual work	% of the time	21.79	30.83	22.05	31.25	20.43	28.52	0.263
Occupational physical activity	from sedentary/standing job to (heavy) manual work	%	16.10	549	16.80	482	12.41	67	0.013
Moderate to vigorous physical activity	<3.5 hours per week	% of the time	20.42	29.17	18.95	28.20	28.27	32.78	<0.001
Moderate to vigorous physical activity	from ≥3.5 hours to <3.5 hours per week	%	33.90	1147	31.79	906	45.22	241	<0.001
Dutch Healthy Diet index 2015	on a scale from 0 – 130	AUE	65.73	11.88	65.84	11.81	65.13	12.22	0.206
Dutch Healthy Diet index 2015	on a scale from 0 – 130	TOE	1.12	6.61	1.12	6.47	1.10	7.29	0.967
Number of hours of sleep	in hours per day (1=≤5, 2=6, 3=7, 4=8, 5=≥9 hours)	AUE	3.27	0.68	3.27	0.64	3.26	0.85	0.647
Number of hours of sleep	in hours per day (1=≤5, 2=6, 3=7, 4=8, 5=≥9 hours)	TOE	-0.11	0.27	-0.11	0.26	-0.10	0.31	0.428
Reproductive cycle status	female, regular cycle	% of the time	21.54	29.68	22.16	30.07	18.24	27.31	0.005
Reproductive cycle status	female, irregular cycle	% of the time	4.16	10.82	3.97	10.41	5.19	12.75	0.016
Reproductive cycle status	female, menopause	% of the time	16.38	27.54	15.61	26.66	20.47	31.52	<0.001
Reproductive cycle status	from regular cycle to irregular cycle or menopause	%	36.06	1232	36.52	1050	33.58	182	0.208
Environmental exposures									
NO_2 concentration	in ug/m^3	AUE	27.67	1.87	27.67	1.86	27.68	1.90	0.922
NO_2 concentration	in ug/m^3	TOE	-1.61	0.62	-1.60	0.61	-1.62	0.65	0.662
$PM_{2.5}$ concentration	in ug/m^3	AUE	20.21	0.61	20.21	0.60	20.21	0.67	0.884
$PM_{2.5}$ concentration	in ug/m^3	TOE	-1.78	0.44	-1.78	0.42	-1.76	0.50	0.227
Elemental carbon concentration	in ug/m^3	AUE	1.32	0.14	1.33	0.14	1.32	0.14	0.679
Elemental carbon concentration	in ug/m^3	TOE	-0.13	0.05	-0.14	0.05	-0.13	0.05	0.651
Rail traffic noise levels	in dB	AUE	31.70	7.16	31.79	7.18	31.18	7.07	0.071
Rail traffic noise levels	in dB	TOE[1]	0.01	1.97	0.00	1.97	0.11	1.96	0.235
Road traffic noise levels	in dB	AUE	52.47	5.09	52.38	5.04	52.95	5.37	0.018
Road traffic noise levels	in dB	TOE[1]	-0.11	1.48	-0.11	1.45	-0.09	1.66	0.764
NDVI in 300 meters buffer	on a scale from -1 – 1	AUE	0.47	0.11	0.47	0.11	0.46	0.11	0.014
NDVI in 300 meters buffer	on a scale from -1 – 1	TOE[1]	0.00	0.03	0.00	0.03	0.00	0.03	0.772
NDVI in 1000 meters buffer	on a scale from -1 – 1	AUE	0.51	0.09	0.51	0.09	0.51	0.09	0.407
NDVI in 1000 meters buffer	on a scale from -1 – 1	TOE[1]	0.00	0.02	0.00	0.02	0.00	0.02	0.283
Damp stains in the house	occasionally or often or always	% of the time	22.22	34.29	21.57	33.94	25.81	35.97	0.014
Damp stains in the house	from not at all to occasionally or often or always	%	9.68	295	9.53	246	10.54	49	0.553
Mold growth in the house	occasionally or often or always	% of the time	11.16	25.44	10.98	25.26	12.15	26.42	0.361
Mold growth in the house	from not at all to occasionally or often or always	%	7.45	227	7.32	189	8.17	38	0.584
Hot water supply in the house	combi boiler	% of the time	51.86	42.68	51.64	42.89	53.09	41.55	0.499
Hot water supply in the house	combination or other	% of the time	16.84	29.93	17.11	30.11	15.35	28.85	0.242

Exposure	Label	Type	Total population (n=3419)		Good perceived health (n=2876)		Poor perceived health (n=543)		
			Mean/%	*SD/n*	*Mean/%*	*SD/n*	*Mean/%*	*SD/n*	*p-value*
Hot water supply in the house	from geyser to combi boiler	%	8.96	274	8.34	216	12.37	58	0.242
Heat source for cooking	gas	% of the time	69.10	42.50	69.03	42.47	69.51	42.72	0.007
Heat source for cooking	from gas to electric	%	6.80	208	7.18	186	4.69	22	0.821
Pet in the house	yes	% of the time	49.10	44.48	49.15	44.50	48.82	44.43	0.061
Pet in the house	from yes to no	%	12.54	383	12.76	330	11.32	53	0.885
Smoking in participant's environment	yes, at home and/or at work	% of the time	46.27	44.32	45.37	44.33	51.28	43.96	0.431
Smoking in participant's environment	from no to yes	%	6.24	191	6.25	162	6.18	29	0.008
Social support (positive experiences)	on a scale from 8 – 32	AUE	22.96	3.10	23.09	3.06	22.24	3.19	1.000
Social support (positive experiences)	on a scale from 8 – 32	TOE	0.41	2.17	0.40	2.15	0.44	2.32	<0.001
Social support (negative experiences)	on a scale from 8 – 32	AUE	12.74	2.36	12.60	2.31	13.49	2.51	0.728
Social support (negative experiences)	on a scale from 8 – 32	TOE	0.25	1.77	0.27	1.69	0.16	2.16	<0.001
Social support measure for elderly	on a scale from 12 – 48	mean r5	30.51	5.60	30.66	5.55	29.61	5.78	0.192
Loneliness scale	on a scale from 0 – 11	mean r5	2.17	2.73	1.94	2.54	3.51	3.31	<0.001
Biological exposures									
Body mass index	in kg/m^2	AUE	25.62	3.51	25.38	3.29	26.85	4.28	<0.001
Body mass index	in kg/m^2	TOE	0.65	0.70	0.62	0.65	0.82	0.90	<0.001
Waist/hip ratio	ratio	AUE	0.90	0.08	0.89	0.07	0.91	0.08	<0.001
Waist/hip ratio	ratio	TOE	0.02	0.02	0.02	0.02	0.02	0.02	0.021
Waist circumference	in centimeters	AUE	92.69	10.85	92.00	10.40	96.38	12.40	<0.001
Waist circumference	in centimeters	TOE	2.16	2.61	2.06	2.53	2.71	2.96	<0.001
Pulse rate	in beats per minute	AUE	70.68	7.29	70.55	7.33	71.33	7.05	0.022
Pulse rate	in beats per minute	TOE	-0.93	3.30	-0.90	3.24	-1.08	3.60	0.233
Systolic pressure	in mm Hg	AUE	125.37	12.98	124.94	12.95	127.61	12.97	0.011
Systolic pressure	in mm Hg	TOE	2.82	4.74	2.77	4.66	3.08	5.15	0.158
Diastolic pressure	in mm Hg	AUE	79.41	7.76	79.27	7.78	80.15	7.57	0.015
Diastolic pressure	in mm Hg	TOE	1.10	3.20	1.17	3.09	0.74	3.70	0.004
Total cholesterol	in mmol/l	AUE	5.50	0.83	5.49	0.83	5.53	0.84	0.350
Total cholesterol	in mmol/l	TOE	0.07	0.31	0.08	0.31	0.00	0.34	<0.001
HDL cholesterol	in mmol/l	AUE	1.39	0.35	1.40	0.35	1.34	0.33	<0.001
HDL cholesterol	in mmol/l	TOE	0.04	0.08	0.04	0.08	0.03	0.08	0.002
Total/HDL cholesterol ratio	ratio	AUE	4.24	1.27	4.21	1.27	4.42	1.26	<0.001
Total/HDL cholesterol ratio	ratio	TOE	-0.07	0.37	-0.06	0.36	-0.11	0.45	0.010
Use of high blood pressure medication	yes	% of the time	9.63	20.94	8.53	19.94	15.49	24.80	<0.001

Exposure	Label	Type	Total population (n=3419)		Good perceived health (n=2876)		Poor perceived health (n=543)		
			Mean/%	*SD/n*	*Mean/%*	*SD/n*	*Mean/%*	*SD/n*	*p-value*
Use of high blood pressure medication	from no to yes	%	17.82	609	16.07	462	27.12	147	<0.001
Use of cholesterol lowering medication	yes	% of the time	5.22	13.94	4.77	13.58	7.59	15.52	0.015
Use of cholesterol lowering medication	from no to yes	%	12.06	412	10.96	315	17.90	97	0.007

AUE, Area-Under-the-Exposure; NDVI, Normalized difference vegetation index; TOE, Trend-Of-the-Exposure
P-values were tested using the independent samples t test and chi-square test.
[1] The noise and NDVI estimates are based on the year 2016 and 2010, respectively. The trend values for these estimates only change when participants change home addresses.

Supplementary Text S3. Prediction performance of the RF model

The total dataset was divided into a training dataset (n=2736) and test dataset (n=683). The AUC of the RF model with the optimal tuning parameters (mtry=10, ntree=1000, nodesize=7, maxnodes=default (n=2736)) for predicting self-perceived health on the training dataset was 0.742. Fitting this model on the test dataset resulted in a slightly lower AUC of 0.707 (95% confidence interval: 0.655-0.759). Without tuning of the parameters (default settings: mtry=9, ntree=500, nodesize=1), the AUC would have been 0.697 (95% confidence interval: 0.644-0.749).

To study which domain(s) of exposures contributed the most to predicting self-perceived health, the relative contribution of a complete domain of exposures (i.e. demographic, lifestyle, environmental, and biological exposures) was also studied. To this end, the prediction performance metrics (AUC, optimal threshold, and sensitivity and specificity at a predefined point) after excluding a particular domain of exposures from the RF were assessed. Thus, metrics were calculated for four different models: one without demographic exposures, one without lifestyle exposures, one without environmental exposures, and one without biological exposures.

Supplementary Table S3 shows all the prediction performance metrics for the total model as well as those for the models without a particular domain of exposures. The AUC values for the model without demographic exposures (0.684), lifestyle exposures (0.695), environmental exposures (0.702), and biological exposures (0.669) were of similar size. Although the model without biological exposures had the lowest maximum of sensitivity plus sensitivity at the optimal threshold (1.256) and the lowest specificity and sensitivity at the predefined point of 0.5 (0.730 respectively 0.685), these prediction performance metrics did not differ substantially from the other models.

Supplementary Table S3. Prediction performance metrics for the total model and the models without a particular domain of exposures

Model	*Optimal threshold ROC curve*						*Sensitivity and specificity at a predefined point of 0.5*	
	AUC (95% CI)	threshold	specificity	sensitivity	sensitivity + specificity	accuracy	specificity	sensitivity
Total	0.707 (0.655-0.759)	0.789	0.725	0.593	1.318	0.704	0.777	0.787
Without demographic exposures	0.684 (0.630-0.739)	0.792	0.713	0.565	1.278	0.690	0.767	0.759
Without lifestyle exposures	0.695 (0.642-0.747)	0.875	0.494	0.815	1.309	0.545	0.774	0.806
Without environmental exposures	0.702 (0.650-0.754)	0.866	0.539	0.796	1.335	0.580	0.774	0.796
Without biological exposures	0.669 (0.611-0726)	0.811	0.645	0.611	1.256	0.640	0.730	0.685

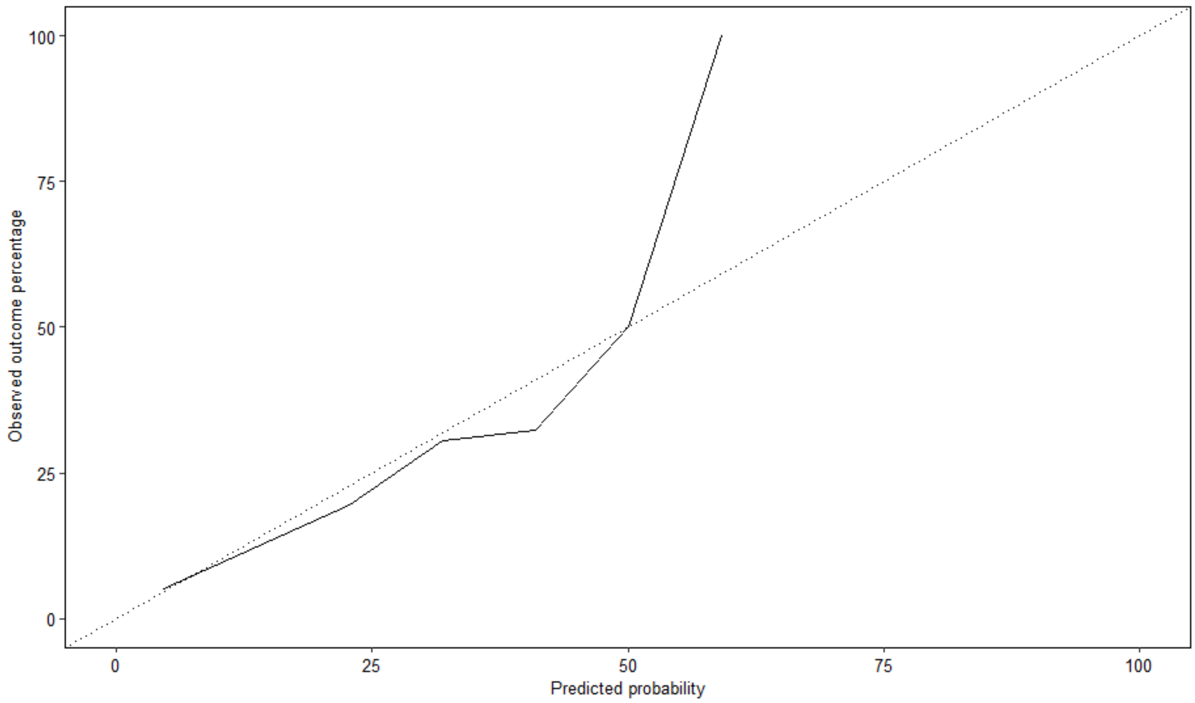

Supplementary Figure S2. Calibration curve showing the predicted probabilities and the observed outcome (i.e. poor self-perceived health) percentages in the test dataset. The dotted line represents perfect calibration. None of the participants had a predicted probability of 60% or higher, and only one participant was included with a predicted probability >55%, explaining the spike at the end of the line.

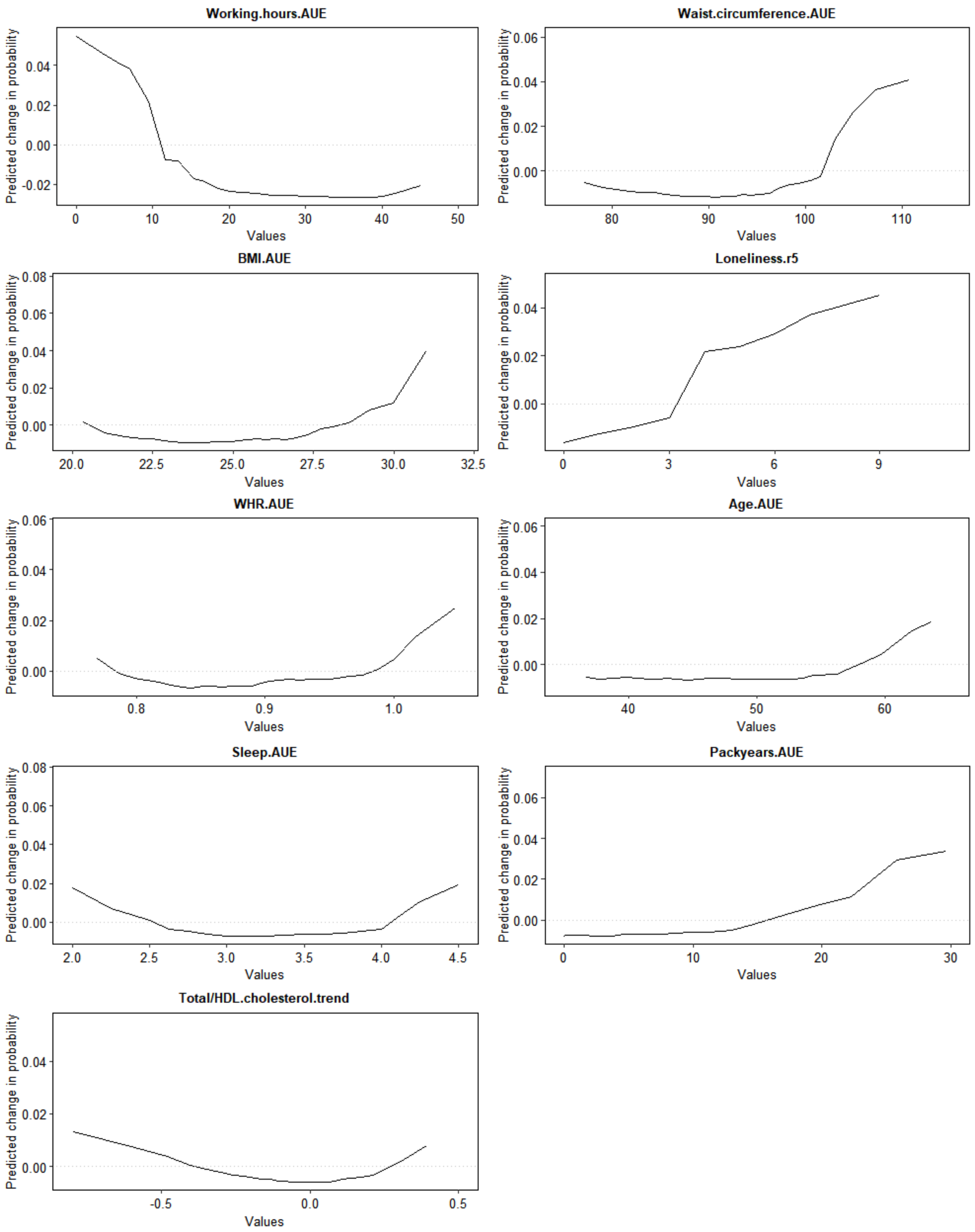

Supplementary Figure S3. Accumulated local effects plots of the relation between predictors of self-perceived health and poor self-perceived health. The dotted gray line represents the reference value, i.e. the local effect is 0. Sleep.AUE is the sleep duration in hours per day where 1=≤5, 2=6, 3=7, 4=8, and 5=≥9 hours of sleep. AUE, Area-Under-the-Exposure; BMI, body mass index; r5, round 5; WHR, waist/hip ratio.

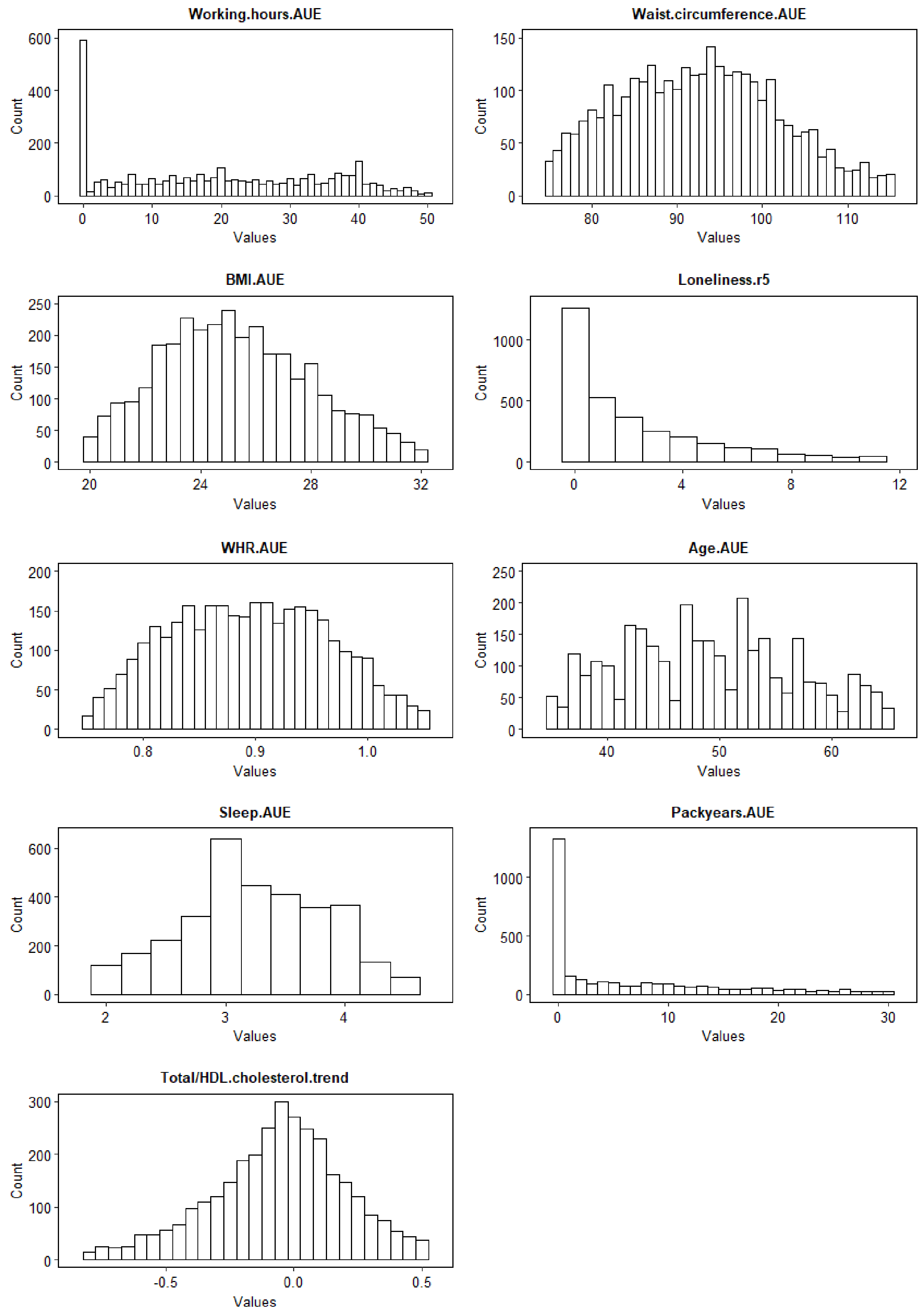

Supplementary Figure S4. Distribution of the values of the predictors of self-perceived health. Sleep.AUE is the sleep duration in hours per day where 1=≤5, 2=6, 3=7, 4=8, and 5=≥9 hours of sleep. AUE, Area-Under-the-Exposure; BMI, body mass index; r5, round 5; WHR, waist/hip ratio.
